# The study on surface morphology and decorative properties of Magnesium-glass-board (MGB)

**DOI:** 10.1371/journal.pone.0296288

**Published:** 2024-01-29

**Authors:** Chaojun Song, Jinxin Wang, Zhanwen Wu, Feng Zhang, Zhaolong Zhu, Xiaolei Guo, Pingxiang Cao

**Affiliations:** 1 College of Materials Science and Technology, Nanjing Forestry University, Nanjing, Jiangsu, China; 2 College of Furnishings and Industrial Design, Nanjing Forestry University, Nanjing, Jiangsu, China; Federal University of ABC, BRAZIL

## Abstract

In order to improve the decorative properties of Magnesium-Glass-Board (MGB), the surface morphology and decoration performance of MGB, are studied in detail by using profilometer, microscope and SEM, and the influence of its characterization, such as surface roughness, surface porosity and wettability, on decorative properties of MGB is analyzed by comparing with medium density fiberboard (MDF) and medium density particleboard (MDP). The results first showed that the surface of MGB has a porous structure, but MDF and MDP are not, resulting in a poor decorative performance of MGB. Second, it is found that the surface wettability of MGB is better than others. Third, the hot-pressing parameters including pressure, temperature and time have different influence on decorative performance of MGB during hot-pressing experiment. Finally, the surface bonding strength is positively correlated with pressure, but not with temperature and time. In general, a higher surface bonding strength led to a better decorative performance of MGB. The furthermore research can concentrate on the modification method of the MGB’s surface according to this paper’s conclusion to improve the lamination performance of melamine paper.

## 1. Introduction

Magnesium-glass-board (MGB) is a kind of excellent fire proof material, which is water-proof, moisture-resistant, odourless, non-toxic and light [1]. MGB also has the advantage of high strength, convenient construction, long service life, etc. The production process of MGB is relatively simple, which neither produces any waste gas and waste liquid difficult to deal with, nor produces any harmful solid waste [2]. Slag, construction waste and other solid waste can also use as filler material, which not only reduces waste but also can bring economic value [3]. MGB can be used as ceilings, wall boards and floorboards and also can replace artificial boards employed as doors and windows, furniture, etc [4, 5]. It will have a certain positive significance to the development of green building materials and ecological civilisation in worldwide if the further application of MGB is promoted in the domestic market.

In interior decoration, it was not enough to only consider the performance and strength of materials. Appearance and color were also important factors affecting people’s quality of life. On the one hand, with the development of productive forces, Aesthetic needs of people had become higher. People needed to get more aesthetic feedback from interior decoration. On the other hand, color and texture could affect people’s emotional feelings. This puts higher requirements for the appearance of the plate. As a basic intermediate material, surface decoration is an important factor of its popularization and application.

MGB is a basic intermediate material, which must be decorated on the surface in order to increase the added value of utilization [6]. Nowadays, although many domestic enterprises have used melamine impregnated adhesive film paperto decorate the surface of MGB [7], there are generally problems of low production efficiency and high defective rate, thus to a certain extent affecting the application of MGB in the domestic market.

After a long period of research by scholars, the raw materials for the base material of veneer panels have been expanded to a larger extent [8–10]. On the basis of traditional man-made panels, other non-biomass materials such as plant straw [11], bagasse or Agro-industrial by-product shells have been developed [12, 13]. In process research, scholars often start with the hot-pressing process parameters and investigate the effects of hot-pressing pressure, hot-pressing temperature and hot-pressing time on the surface bonding strength and mechanical properties of veneer panels [14, 15]. Based on the experimental results, the optimum hot-pressing parameters are investigated for the substrates used in the tests, or for the modification of the substrates with certain materials. These studies have been effective in optimizing the production process and improving the mechanical properties of veneer panels. In addition to this, Demir Aydin and Aydin Ismail have found that the surface bonding strength of veneer panels is closely related to the contact angle and surface free energy of the veneer [16]. Veneers with lower contact angles and smoother surfaces have higher surface free energy values, and plywood made from veneers with higher surface free energy tends to have higher bonding strength. The surface properties of the substrate have been explored in relation to the hot-pressing process and bonding strength of veneers.

Based on the research mentioned above, the low efficiency and high defective rate of MGB surface decoration are still desirable to solve for extending the real-world application of MGB material. In order to solve these problems, it was necessary to analyze the surface properties and decorative properties of MGB. The research was studied by two ways. Firstly, the results of surface roughness, porosity and water contact angle of MGB were compared with that of medium density fiberboard (MDF) and medium density particleboard (MDP) to analysis its surface performance [17–20]. Second, the hot-pressing test was performed to further investigate the decorative properties of MGB. This objective of this study is to provide the mechanism of MGB surface decoration performance.

## 2. Material and experiment design

**Fig 1:**
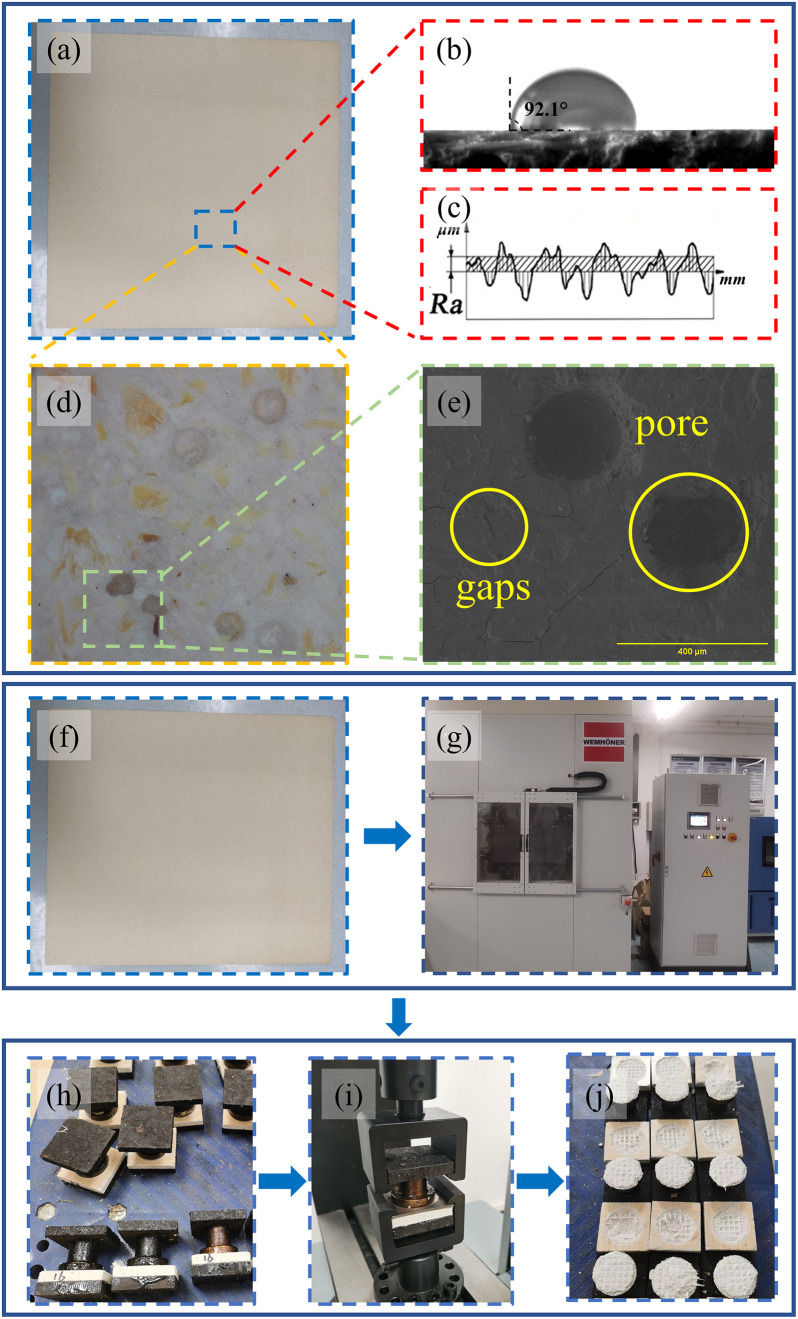
Experimental Flow Organisation (a) magnesium glass board (MGB) for surface test; (b) the measure of contact angle; (c) the measure of surface roughness (Ra); (d) the cached surface images; (e) the SEM images; (f) the workpieces for Surface decoration test; (g) hot press machine (Wemhöner); (h) the test specimen for measuring bonding strength; (i) mechanical testing machine; (j) the specimen after test.

The experimental process and materials of this study was shown in Fig 1. MGB, MDF, MDP and Melamine impregnated adhesive film paper were materials used in this experiment and supplied by Jiangsu Jin Peng Fire Prevention Board Industry Co., Ltd. At the beginning, all those materials need to be cut into standard test panels of 500 × 500 mm (Fig 1a and 1f). The various properties of the panels were showed in the Tables 1 and 2.

**Table 1:** The physical properties of workpieces.

Workpieces	Density (g/m^3^)	Moisture content (%)	Wet expansion rate (%)	Dry shrinkage (%)
MGB	1.12	10.30	0.19	0.12
MDF	0.55	7.13	0.58	0.40
MDP	0.48	7.90	1.61	0.75

**Table 2:** The physical properties of melamine impregnated adhesive film paper.

Dipping amount (%)	volatile content (%)	Precuring degree (%)
205	7.4	42

The observation of surface morphology was carried out through two ways. A microscope (ZW H1600, China Micro Semiconductor (Shenzhen) Co., Ltd, China) and an environmental scanning electron microscope (quanta 200, FEI) were employed for macroscopic morphology (Fig 1d) and microscopic morphology (Fig 1e), respectively. Then the results were compared.

The JB-4C (Shanghai Taiming Optics Co., Ltd., Shanghai, China)) surface profilometer was used to measure the surface roughness (Fig 1c) of MGB, MDF and MDP in order to make an analysis of the surface unevenness. Regarding the effectiveness of experiments results, four independent specimens of MGB were tested, and a total of 20 tests were conducted.

The surface porosity was observed on both sides of the MGB by quanta 200 environmental scanning electron microscope, with a magnification of 40x selected. The pores or gaps which were not clearly visible in the image at this magnification and appear dark black in colour were defined as pores, and the proportion of the pore area to the total area of the image at this magnification was taken as the surface porosity, which was used to quantitatively describe the porosity of the material surface. The porosity was evaluated by the analysis of binary image resulted from the sweep electron microscope. Five randomly positioned images of each side were taken for analysis, using ImageJ software and machine vision learning to implement this process.

In order to directly study the surface decoration performance of MGB, the single-side hot-pressing test of MGB was carried out by using an experimental hot press (Fig 1g, LAP Press, Wemhöner, Changzhou, China). The parameter settings of the MGB veneer test were shown in Table 3. The change of glue was the main content of hot-pressing. The substrate has little influence on this process. Therefore, the parameters of selection were commonly used in hot-pressing of other wood-based panels.

**Table 3:** Parameter settings of the MGB veneer test.

No.	Pressure(MPa)	Temperature(°C)	Time(s)
1	2.0	150	120
2	2.0	160	150
3	2.0	170	180
4	2.5	150	150
5	2.5	160	180
6	2.5	170	120
7	3.0	150	180
8	3.0	160	120
9	3.0	170	150

Then the tests were carried out in accordance with the method specified in GB/T 17657–2013 "Test Methods of Evaluation the Properties of Wood-based Panels and Surface Decorated Wood-based panels". After hot-pressing, the MGB was cut into 50×50 (length × width mm) specimens, and a circular groove with an inner diameter of 35.7 mm and a depth of 0.3 mm was milled at the centre of the specimen. After removing the dust, the specimens were placed at a constant temperature and humidity box with a temperature of (20 ± 2) °C and a relative humidity of (65 ± 5) %. The circular surface of the specimen was glued to the groove of the mechanical testing machine by hot melt glue. And the glue was not allowed to overflow into the groove (Fig 1h). When completely cooling, the specimen was clamped (Fig 1i). The load was increased uniformly to break the specimen in (60±30) seconds (Fig 1j), the maximum load during this process was noted down to 1 N and the bonding strength of the specimen was calculated according to Eq (1).

$$\sigma =\frac{F_{max}}{A}$$ (1)

where σ (MPa) is surface bonding strength of workpieces, Fmax(N) is maximum load at damage to the workpieces, A is the bonding area of the test piece to the chuck set as 1000 mm^2^.

In recent years, trainable machine learning has become a powerful tool for image processing, which can help to make appropriate processing for the images of MGB surfaces. Trainable Weka Segmentation can combine the powerful image processing software Fiji and machine learning algorithms from the Waikato Environment for Knowledge Analysis toolkit to automatically recognize boundaries, textures, and membrane structures in images, transforming the image recognition problem into a pixel classification problem. The remaining pixels were classified by learning user-defined features until a satisfactory outcome was achieved. With this tool, this paper studies the porosity problem on the surface of MGB intuitively, accurately, and effectively.

A fully automatic single fibre contact angle measuring instrument, OCA40 (Dataphysics company, German), was used to test the water contact angle (Fig 1b) on both sides of the MGB and the surface of the MDF and MDP in order to comparatively study the wetting performance of the MGB and the surface of the MDF and MDP. During the test, 5 different locations were taken for each surface tested.

## 3 Results and discussion

### 3.1 Surface morphology

**Fig 2:**
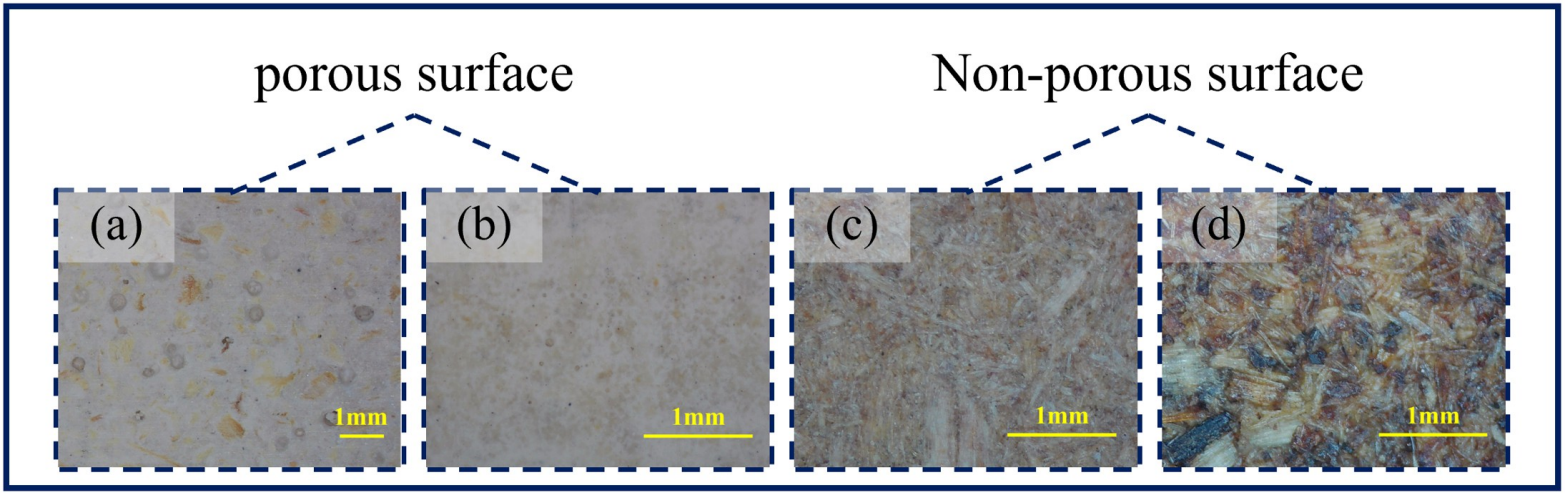
The surface morphology of the workpieces, where (a) was the glossy side of MGB; (b) was the rough side of MGB; (c) MDF and (d) MDP.

The results of macroscopic morphology of the surfaces of MGB, MDF and MDP were showed in Fig 2. The smooth surface still had many pores showed in Fig 2(a), but they were relatively small and needed to be observed carefully. As shown in Fig 2(b), the rough surface of the MGB was relatively flat and smooth, but there were many large pores can be observed. MDF had a fine and even surface. The dark dots were woody material rather than pores in Fig 2(c), and it had a little bit blocked feel when touching. But it still had a smooth surface condition. MDP had the largest visual surface component unit and the greatest surface roughness, but it was more completely filled and did not have visible pores in Fig 2(d).

A comparative analysis showed that the main difference between the surface of MGB and MDF and MDP was that the surface of MGB was less well-filled and had more porous. The surface of MDF and MDP was completely filled and did not have obvious pores. Apart from the porous part, the surface of MDF and MDP was relatively rough.

**Fig 3:**
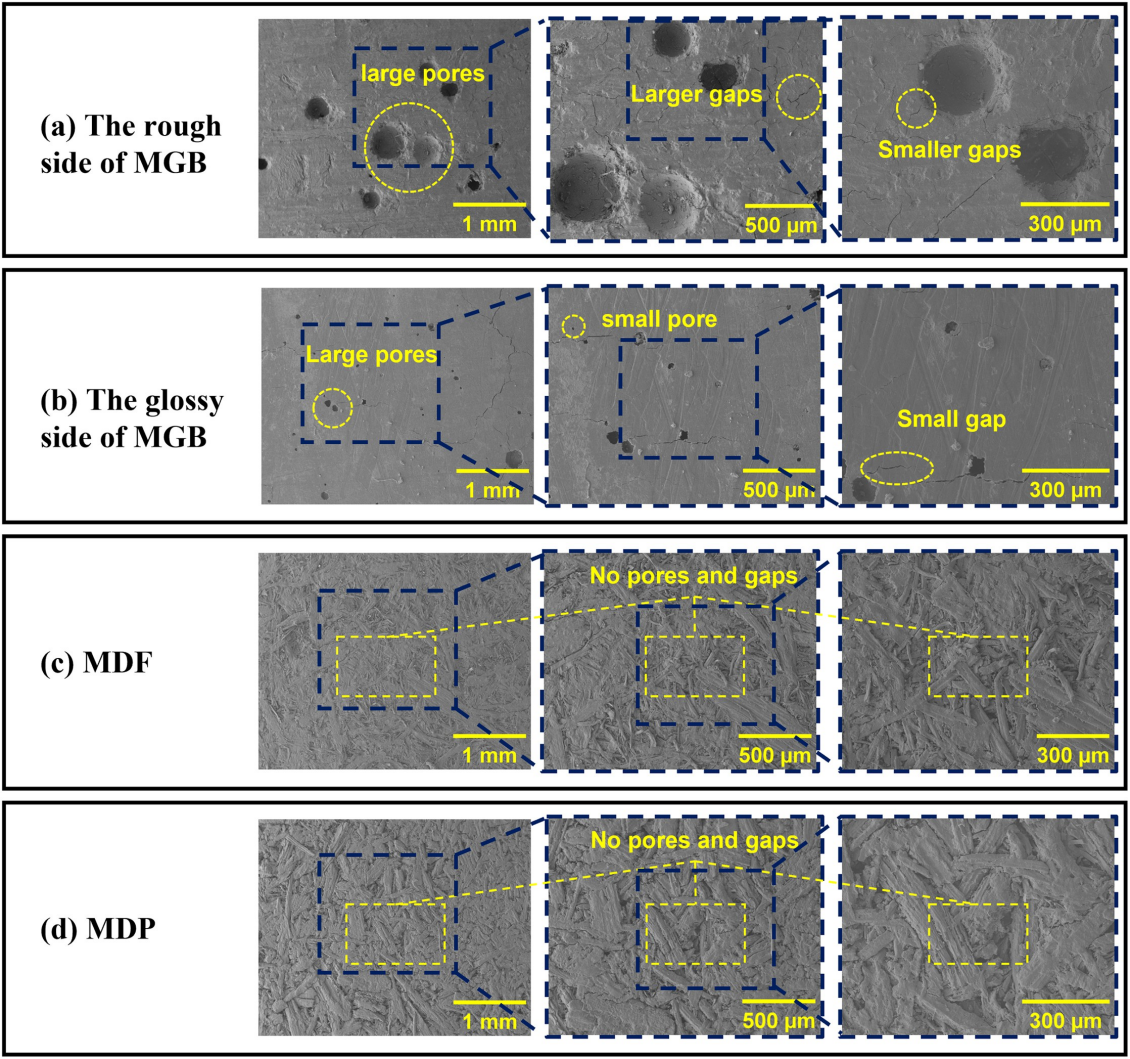
The SEM images of the three types of workpieces at different magnifications: (a) the rough side of MGB; (b) the glossy side of MGB, (c) MDF and (d) MDP.

The results of microscopic morphology of the surfaces of MGB, MDF and MDP were showed in Fig 3. The surface morphology of the rough and glossy surfaces of the MGB was relatively similar, both consisting of flat and glossy planes and pores, and there was a certain number of gaps. As shown in Fig 3(a), the rough surface of MGB was lightly flat, and it had larger size of pores in general; the glossy surface was flatter in Fig 3(b), and the pores were generally smaller in size. The surface morphology of MDF and MDP was similar, a multi-layer structure was observed. One layer was consisted of fibres or particles. That structure caused a solid composite with fewer pores. Furthermore, both of them were formed by the stacking and pressing of fine bulk fibres or shavings.

According to the surface morphology observation of those materials, a further analysis of the hot-pressing process of them was given as follows. When MDF and MDP were pressed and laminated, the leakage process of the glue from the gaps between the adjacent fibres or shavings would be blocked by the underlying fibres or shavings until curing. Therefore, all glues were hard to flow away, causing a better gluing effect. On the other hand, the uneven surface of MDF and MDP can create anchoring conditions for the glue nails formed by the glue solution, which facilitates a tight bond between the adhesive film paper and the MDF or MDP surface. Therefore, MDF and MDP were more suitable for pressing and decorating. In contrast, the surface of MGB was very glossy and the glue does not create a stable anchoring effect. At higher hot press pressures, the glue was more likely to flow into the pores, making the bonding effect in a poor situation.

### 3.2 Surface roughness

The surface roughness value Ra of MGB and MDF and MDP was tested to evaluate the surface quality, and the test results were showed in Table 4. This showed that the height difference between the pore and MGB surface was large, which lead to a large dispersion of Ra. Some results had larger values, indicated that some pores had larger depths. The results of MDF and MDP showed a similar Ra, indicating that there were no obvious pores on the surface of MDF and MDP or the pores were smaller or less. But the Ra value of MDP was less than that of MDF, it was likely because the area of particle was larger than that of the wood fibres, and the travel of the stylus in that area was shorter than that of the MDF. That caused a better surface quality of MDP. Besides, the difference of Ra between MGB and MDF or MDP can be organised as follows. There were more grooves on the surface of the MDF. That was beneficial for the glues bonded between the impregnated paper and the substrate.

**Table 4:** The surface roughness of the three types of workpieces.

Workpieces	Point 1	Point 2	Point 3	Point 4	Point 5	Average
The rough side of MGB	4.305	1.628	14.822	3.415	4.177	5.670
	1.163	1.251	7.340	1.493	8.590	3.967
	1.289	1.096	7.403	0.864	3.034	2.772
	6.161	1.461	2.742	3.976	7.605	4.389
The glossy side of MGB	0.632	3.863	4.006	0.674	0.581	1.951
	0.470	0.276	0.617	0.338	0.507	0.442
	3.044	0.314	0.305	0.489	0.355	0.901
	0.628	0.811	0.885	1.004	2.152	1.096
MDF	4.375	5.822	5.207	4.750	3.654	4.762
MDP	3.724	4.183	2.720	6.272	3.806	4.141

### 3.3 Surface porosity

The analysis of the structural composition of MDF and MDP shows that their surface pores is shallow, and is almost completely fil by adhesive. So, in this context, MDF and MDP have almost no surface porosity. Therefore, only the surface porosity of both sides of the MGB is analyzed here. The analysis of the structural composition of both showed that the depth of surface pores was shallow, therefore only the surface porosity of both sides of the MGB was analyzed here. Five randomly positioned images from each side were taken for analysis, using ‘Image J’ software and machine vision learning to implement this process [21, 22].

**Fig 4:**
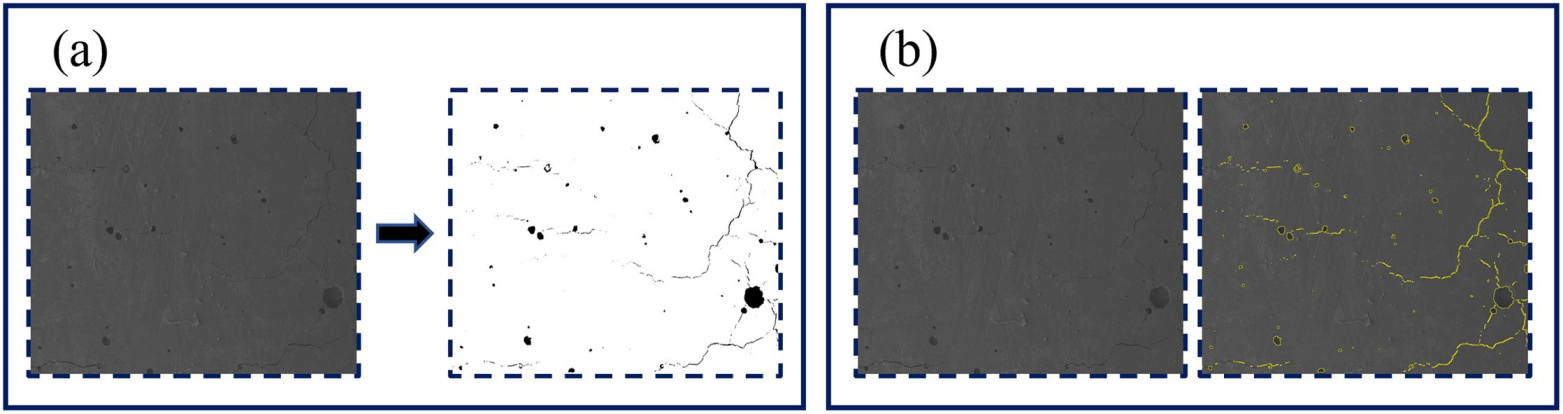
The identification and contrary with surface voids of MGB using machine learning.

Machine vision learning was carried out in this study using the Trainable Weka segmentation plug-in [23]. Before the surface porosity of the MGB was calculated, a porosity recognition model was first trained. A few porosity sections in the SEM images of the surface of the MGB were selected and defined as Class 1, followed by a few sections of the surface of the MGB positioned as Class 2 to start training the machine vision recognition model. After the model was trained, it was compared with the SEM image without recognition to correct the training results. This procedure was repeated until good results are obtained. The final effect of pore recognition was shown in Fig 4. Fig 4(a) showed the identification results of pores, while Fig 4(b) showed a comparison of the effectiveness of air raid identification. The recognition results showed that this method has a high accuracy in identifying the surface pores of MGB, and further statement will be conducted on this basis.

**Fig 5:**
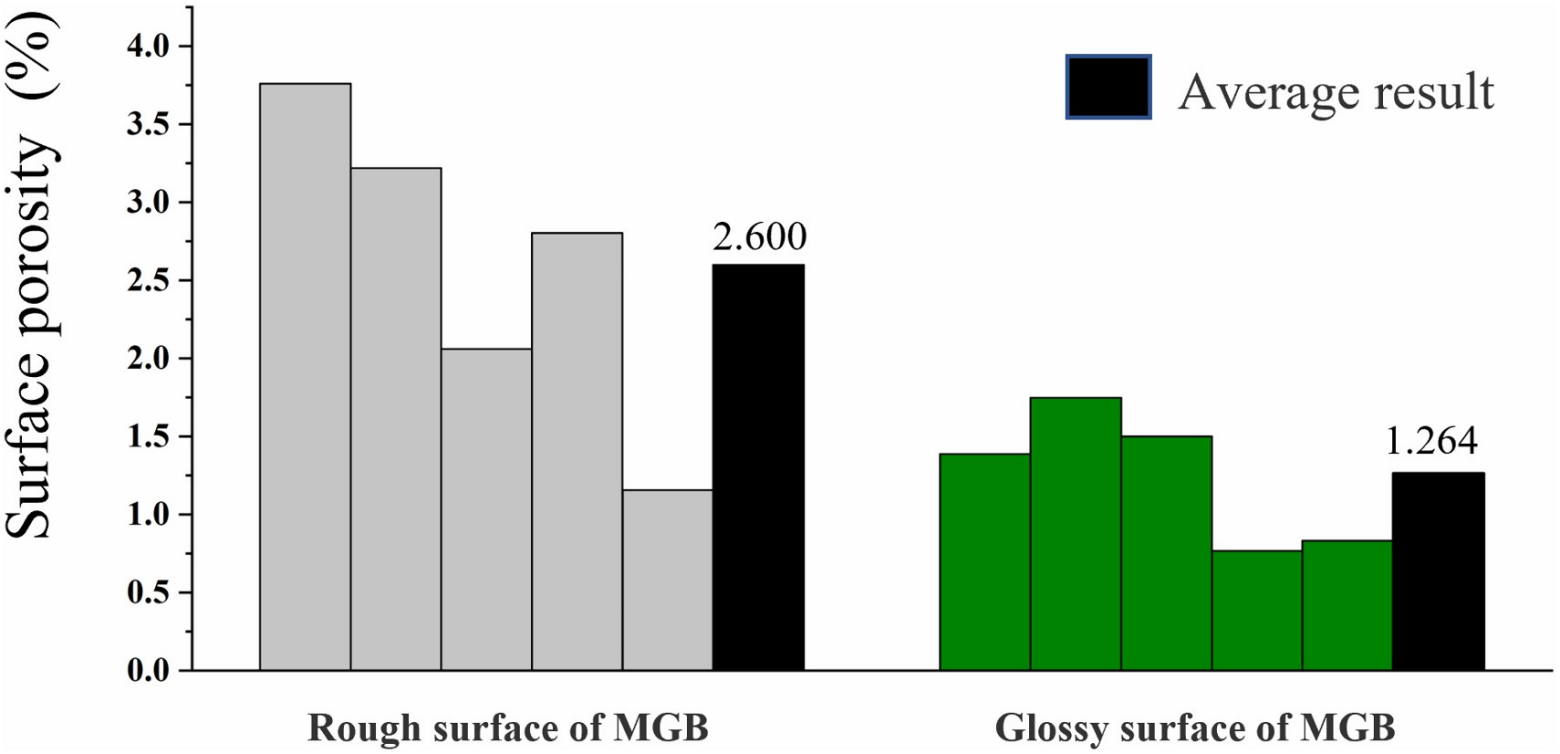
Surface porosity of MGB.

The results of surface porosity were showed in Fig 5. It can be seen that the surface porosity of the rough surface of MGB was larger than the smooth surface. However, in actual production, the rough surface of the MGB has a better decorative performance. But, there were many uneven structures and micro-cracks on the rough surface. In contrast, the smooth surface did not have uneven structures, and the gaps were generally larger. Besides, there were almost no micro-cracks observed.

The conclusions can be draw twofold: 1) the uneven structure and micro-cracks on the rough surface of the MGB facilitate the formation of gluing effect. Therefore, it strengthened the bonding strength of the MGB with melamine impregnated adhesive film paper [24]. The number of pore was less on the smooth surface, and it coupled with the uneven structure to prevent the flow of glue to the pores. Thus, leading to a relatively good hot-pressing performance.

### 3.4 Water contact angle

**Fig 6:**
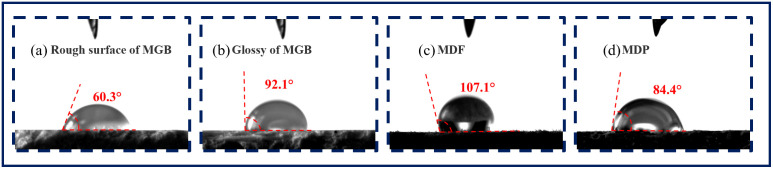
One result of the test of MGB, MDF and MDP.

**Fig 7:**
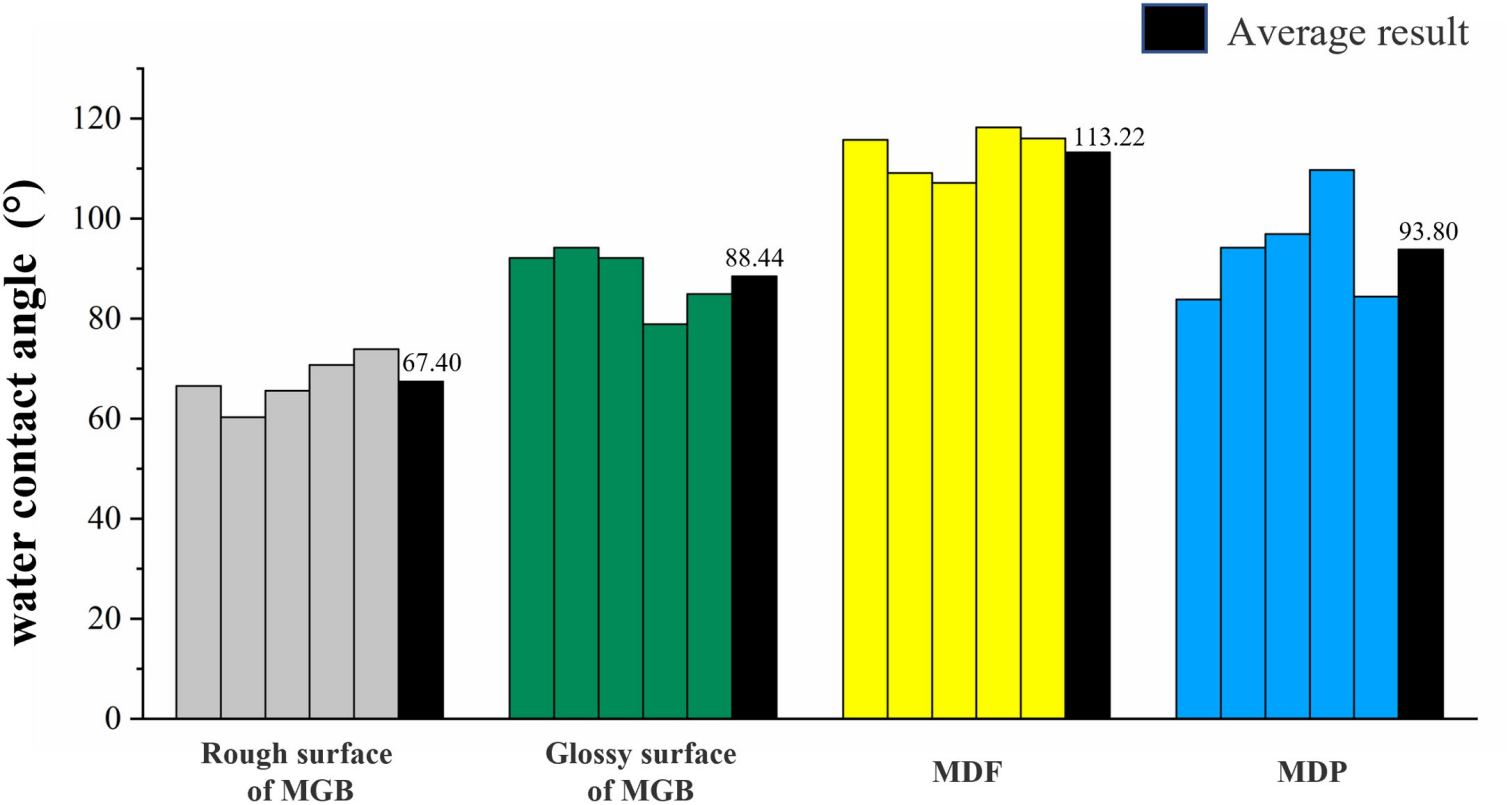
The contact angle of three types of workpieces.

The results of the water contact angle of MGB, MDF and MDP were showed in Figs 6 and 7. Fig 6 showed one result of the test of MGB, MDF and MDP. All results and average values of the test were showed in Fig 7.

As can be seen from Fig 7: the water contact angle on both sides of the MGB was less than 90°, proving that both surfaces of the MGB had good wetting performance [25]. The water contact angle of both MDF and MDP was greater than 90°, which means that the wetting performance of both surfaces was poor. The relationship between the water contact angle of the two surfaces of MGB and MDF and MDP was: the rough surface of MGB < the smooth surface of MGB < MDP < MDF. It is likely that the more surface Oxygen bonds can make the surface hydrophilic and lead to a lower water contact angle.

The wetting properties of the board surface had a significant influence on the flow of the glue during hot-pressing. Compared to MDF and MDP, the surface of MGB has better wetting properties [26], making it easier for the glue to flow during hot-pressing. The pores on the surface of the MGB were where the glue flows easily. Under the high pressure of hot-pressing, the glue around the pores can easily flow to the pores on the surface of the MGB, resulting in a larger area of glue loss, giving rise to the lack of glue at the voids of the MGB, making the surface of the MGB at the pores of the gluing effect poor. The direction of the pores on the surface of the MGB was vertical board direction, the water vapors generated inside the board when hot-pressing was more likely to permeate from the surface of the impregnated paper, making the board appear bulging or even cracking quality problems when installed.

The main process of surface decoration of MGB was that melamine resin melts and flows at a certain temperature, then solidifies at a higher temperature. So that MGB substrate and impregnated paper were closely combined. In this process, if the surface morphology of MGB is too rough or Ra is too large, the impregnated paper may not be closely attached to the surface of MGB, thus reducing the bonding strength. The wettability of MGB surface mainly affects the flow of melamine resin. If the surface wettability of the substrate is not good enough, the melamine resin can’t fully flow. It is likely that the melamine resin fails in filling the large gap between the impregnated paper and MGB, leading to a lower bonding strength. In general, the surface morphology, roughness and wettability can affect the bonding strength between MGB substrate and impregnated paper.

Based on the analysis of surface roughness, surface porosity and water contact angle, the main findings were presented as follows: (1) the difference of surface roughness among them was small, so it was not the main reason that affected the decorative performance of MGB; (2) MGB had a high surface porosity. It was the main factor that affects its decorative properties. It was also the key factor improve the decorative properties of MGB; (2) the wettability of MGB was better than MDF and MDP. If the surface of MGB was non-porous, it would be beneficial to decorative performance. Therefore, reducing the surface porosity of MGB was the main method to improve its decorative properties.

### 3.5 The factors influencing the bonding strength of impregnated paper veneers

The parameter settings and results of the MGB veneer test were shown in Table 5. Due to the fact that one side of MGB was relatively smooth and can be directly used, this study used a single side hot-pressing method to treat the rough side of MGB. According to GB/T 15102–2017, "Surface decorated fiberboard and particleboard with paper impregnated thermosetting resigns," when the density of the substrate was greater than 0.8g/m3, the surface bonding strength of MDF should be greater than 1.0 MPa, and the surface bonding strength of MDP should be greater than 0.6 MPa. Within the parameter range, the lowest surface bonding strength of the veneered MGB obtained in this paper was 2.56 MPa, much higher than that of the general veneered fiberboard or MDP. The differences in surface roughness, surface porosity, and water contact angle can explain the phenomenon.

**Table 5:** Bonding strength of surface decorated MGB.

No.	Pressure	Temperature	Time	Bonding strength
1	2.0	150	120	2.66
2	2.0	160	150	2.65
3	2.0	170	180	2.77
4	2.5	150	150	2.56
5	2.5	160	180	2.75
6	2.5	170	120	2.87
7	3.0	150	180	3.04
8	3.0	160	120	3.06
9	3.0	170	150	2.68

Although the surface roughness of MDF was similar to the mean value of which of the rough surface of the MGB, in fact, in the substrate of MDF and MDP, the pores between the wood fibers or shavings were roughly filled by the glue solution, the surface morphology was uniform with very few pores. Moreover, the Ra value of the surface of the MGB was combined with the surface of the pores, pits, and small cracks. As a result, the surface topography and the distribution of uneven structures were not uniform. When hot-pressing, this uneven glue adhesion and gluing structures provided a morphological basis; the glue can form a glue nail, and impregnated paper with the MGB substrate, like a boat anchor, was firmly fixed together. At the same time, the water contact angle of the MGB was much lower than that of the MDF and MDP, the glue had better mobility on its surface, and it was easier to move into the uneven structure of the surface of the substrate, to make the anchoring effect play more fully.

Analysis of variance (ANOVA) showed that the influence of the three hot-pressing parameters on the bonding strength of MGB veneer was in the order of pressing time over pressure over temperature (see Table 6). This was because the time of hot-pressing had a significant effect on the drainage of moisture from the MGB. The pores in the MGB were often oriented perpendicular to the board surface. When hot-pressing, the water vapour formed by the vaporisation of the water in the board was more easily discharged along the pores and had an obvious mechanical effect on the laminated impregnated paper, so the impact on the surface bonding strength was most significant [27]; the hot-pressing pressure played a role in the hot-pressing process to promote the uniform flow of the glue, thus forming a more uniform glue film structure. It also reduces the warpage of the substrate and promotes the uniform contact between the impregnated paper and the substrate, so it can also influence the surface bonding strength to a greater extent; the main role of the hot-pressing temperature in the hot-pressing process was to promote the curing of the melamine resin, which had less influence on the hot-pressing process of the MGB and the melamine impregnated adhesive film paper.

**Table 6:** The response of MEAN.

Level	P	T	TIME
1	2.707	2.753	2.863
2	2.727	2.833	2.643
3	2.927	2.773	2.853
Delta	0.220	0.080	0.220
Rank	2	3	1

**Fig 8:**
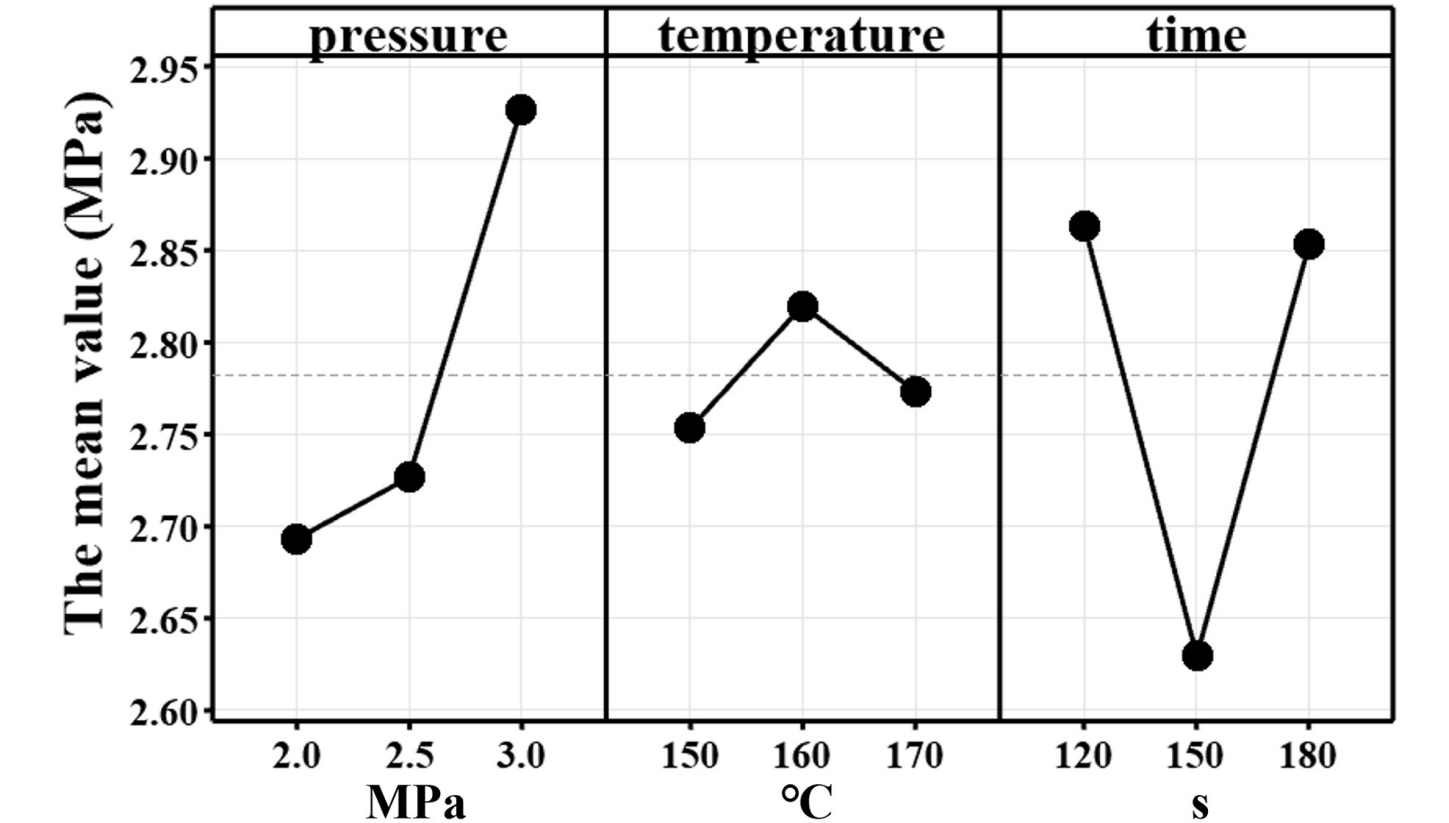
Response diagram of bonding strength versus hot-pressing parameters.

As can be seen in Fig 8, the bonding strength showed a clear positive correlation with pressure, but the correspondence with temperature and time was unclear. As the hot-pressing pressure increased, the glue spread more easily. Making more evenly distributed and easier to form glue nails in uneven areas of the surface. Therefore, the surface bonding strength and pressure showed a positive correlation tendency.

The bonding strength was firstly increased with the temperature increase from 150°C to 160°C in Fig 8. The reason could be that the melamine resin solidifies with the increase of temperature. But, when the temperature continues to increase to 170°C, this higher temperature could boost the whole process. It is likely that the flow time of the resin is not enough to fully fill the gap between the impregnated paper and MGB and thus it could decrease the bounding strength. Therefore, the surface bonding strength showed a tendency to rise and then fall with increasing hot-pressing temperature.

In contrast, as shown in Fig 8, the bonding strength firstly decreased with the increase of hot-pressing time and then increased when the time continues to grow. When the time reached 120s, less water vapour was generated during the hot-pressing process. The mechanical effect on the melamine impregnated adhesive film paper was less, enabling a better gluing quality to be obtained.

When the time increased to 150s, lots of water vapour generated during hot-pressing of MGB and accumulated the pores of the vertical MGB surface. Therefore, the mechanical effect on the surface of the melamine impregnated film paper was greater when the press opened, reducing the surface bonding strength.

However, when the pressing time was extended to 180s, the water vapour produced during the pressing process had more time to escape from the sides through the small gaps in the MGB panel. Therefore, a better surface bonding strength can be obtained again. This explanation can also be verified by the observation of the hot-pressing process.

## 4. Conclusions

This paper compared the performance of MGB with MDP and MDP in terms of surface morphology, surface roughness, surface porosity and water contact angle. The influence of morphology of MGB on the surface bonding strength was investigated. An orthogonal experiment was designed to explore the degree and tendency of influence of hot-pressing parameters on the surface decoration performance of MGB. The main conclusions can be draw as follows:

1. The Ra values of MGB, MDF, and MDP were relatively close, but the surface morphology differed. The surface of the MGB had a porous structure, and the surface porosity of the rough and smooth surfaces were 2.600% and 1.264%, respectively. The surface morphology of MDF and MDP was more uniform than that of MGB, and no porous structure was observed in either of them.
2. The wettability of MGB surface was better than MDF and MDP, which makes the glue flow more easily on MGB surface. However, the surface of MGB was porous, and the glue would flow into the pores from the surface. There would be less glue on the surface of MGB. Therefore, the decorative performance of MBG would be decreasing.
3. The influence of hot-pressing parameters on the surface bonding strength of MGB was hot-pressing time > hot-pressing pressure > hot-pressing temperature. The surface bonding strength positively correlated with hot-pressing pressure within the parameter selection range. However, the relationship between surface bonding strength and hot-pressing parameters was not obvious.
4. In the subsequent research, the author will study the modification method of the MGB’s surface according to this paper’s conclusion to improve the hot-pressing performance. Furthermore, conducting double-sided hot-pressing research to meet the demands of extending the application of MGB for home furnishings.
